# Parasite-mediated predation determines infection in a complex predator–prey–parasite system

**DOI:** 10.1098/rspb.2023.2468

**Published:** 2024-04-24

**Authors:** Ana C. Hijar Islas, Amy Milne, Christophe Eizaguirre, Weini Huang

**Affiliations:** ^1^ School of Biological and Behavioural Sciences, Queen Mary University of London, London, UK; ^2^ School of Mathematical Sciences, Queen Mary University of London, London, UK; ^3^ Department of Mathematics, Swansea University, Swansea, UK; ^4^ Group of Theoretical Biology, School of Life Sciences, Sun Yat-sen University, Guangzhou, People’s Republic of China

**Keywords:** predator–prey–parasite system, trophically transmitted parasite, equilibrium analysis, stochastic dynamics, species coexistence

## Abstract

The interplay of host–parasite and predator–prey interactions is critical in ecological dynamics because both predators and parasites can regulate communities. But what is the prevalence of infected prey and predators when a parasite is transmitted through trophic interactions considering stochastic demographic changes? Here, we modelled and analysed a complex predator–prey–parasite system, where parasites are transmitted from prey to predators. We varied parasite virulence and infection probabilities to investigate how those evolutionary factors determine species’ coexistence and populations’ composition. Our results show that parasite species go extinct when the infection probabilities of either host are small and that success in infecting the final host is more critical for the survival of the parasite. While our stochastic simulations are consistent with deterministic predictions, stochasticity plays an important role in the border regions between coexistence and extinction. As expected, the proportion of infected individuals increases with the infection probabilities. Interestingly, the relative abundances of infected and uninfected individuals can have opposite orders in the intermediate and final host populations. This counterintuitive observation shows that the interplay of direct and indirect parasite effects is a common driver of the prevalence of infection in a complex system.

## 1. Introduction

Food webs are complex networks composed of species and governed by their interactions, whereby consumers feed on resources to gain energy [1,2]. When a species goes extinct, its local network may also disappear from the food web, potentially altering the system’s stability and dynamics [3]. While food webs tend to be robust to random losses, some species may play crucial roles in maintaining the food web integrity and therefore are likely to cause the greatest damage if removed [4]. The degree of impact caused by the loss of a species is characterized by its number of interactions with other species and the degree of specialization of those interactions [3].

Parasites represent large biomass on Earth [5] and are involved in about 75% of food web links [6]. Parasites impact their host population through changes in body condition [7], behaviour [8] and reproduction [9]. Fighting parasite exposure demands mounting an immune response which is energetically costly and, as such, exposure without infection can result in lower reproductive investment and fecundity than unexposed hosts [10–12]. This parasitic outcome has been observed in snail–trematode systems, for instance, and is common in insects infected by protozoans, nematodes and cestodes [10]. The consequences of infection go beyond a single population because hosts are connected with other species, such as predators and prey [13]. In natural systems, parasites can infect multiple hosts directly or via trophic interactions [14]. Trophically transmitted parasites provide natural biological indicators of trophic links because they are the accumulated consequence of long-term feeding by their hosts [15,16], and are common in nature [5]. For example, the tapeworm *Schistocephalus solidus* uses copepods and fish as first and second intermediate hosts, before trophically reaching the final bird host [17]. The nematodes *Camallanus lacustris* and *Anguillicola crassus* are trophically transmitted and use the three-spined stickleback *Gasterosteus aculeatus* as a paratenic host [18]. Some trophically transmitted parasites can even manipulate the behaviour of their intermediate prey host in ways that increase consumption rates, and therefore transmission to their final predator host [19–21]. This flux transfer has consequences on the food web connectance and stability [22]. Despite their ecological importance, however, the mechanisms driving the dynamics of trophically transmitted parasites are not well understood [16].

From an ecological perspective, biological populations exhibit a large spectrum of dynamical behaviour from stable equilibrium points, to stable cyclic oscillations and chaotic dynamics [23]. The latter can refer to the appearance of aperiodic cycles in population dynamics and may occur when the *per capita* rate of increase exceeds some threshold value [24]. A biological system is considered internally stable if it does not experience significant changes in its characteristics and returns to a steady state after a perturbation [23]. Theoretical work on a complex food web model demonstrates that the fluctuations in predator–prey population dynamics change according to the attack rate of the predator [25]. The model shows that too inefficient or too aggressive predators result in vigorous population fluctuations and prevent predator–prey coexistence [25]. In various predator–prey–parasite models, the infection of only predator species [26,27], only prey species [28–31], or both predator and prey species but by different parasites [32], can impact predator–prey population dynamics. The inclusion of parasites in predator–prey systems often leads to chaotic dynamics [33–35] because epidemiological processes (such as disease) can alter the death and birth rates of the predator and prey hosts and lead to aperiodic cycles in predator–prey dynamics. While previous models illustrate the effects of parasitism on predator–prey interactions, they often focus on the infection of single host species (either the prey or the predator) or do not consider stochastic demographic fluctuations [36,37]. Here, we focus on species coexistence and the prevalence of infection in different trophic levels when the parasite mandatorily infects both the prey and the predator under stochastic demographic changes.

Besides classical predator–prey cycles often referred to as total abundances in each species, subpopulations of infected and uninfected hosts coexist at equilibrium [38]. The relative frequencies of infected and uninfected hosts are likely to be relevant in community dynamics. While parasites are not entirely or instantly fatal to their hosts [14], their consumptive effects may result in trait-mediated trophic cascades [39]. When infection is highly detrimental to the prey, a high frequency of infected prey may have a bottom-up effect on the food web, e.g. the reduction of prey species (bottom) is followed by the population decline of its consumers (up) [40]. Also, a high frequency of infected prey increases the probability of parasite transmission to higher trophic levels. When infection is highly detrimental to the predator, a high frequency of infected predators may lead to top-down effects because the reduction of predator (top) abundance relaxes the predation pressures on the prey, allowing the prey (down) to reproduce more rapidly [40]. The resulting increase in prey abundance leads to stronger pressures on lower trophic levels such as smaller prey or plants. These cascading effects can impact community structure and dynamics [41] as well as ecosystem functioning [41,42]. The strength and frequency of parasite-mediated trophic cascades will be determined by the susceptibility of the host to the parasite [42] and the magnitude of fitness costs due to infection [39].

Here, we built an individual-based model to capture such a complex predator–prey–parasite system, based on microscopic events, including reproduction, intrinsic death, competition, infection and predation for relevant species. Note that when applying individual-based modelling in ecological systems, we refer to individuals as microscopic components and populations and communities as macroscopic organizational levels, hence microscopic events refer to individual-level events [43]. In our model, the parasite is transmitted trophically from infected prey to predators [17,18]. We investigated the extent to which different factors such as the infection probabilities and parasite virulence (here defined by the reproductive costs imposed on infected hosts) [10–12,44] affect the transmission and persistence of the trophically transmitted parasite. We decomposed the predator and prey populations into infected and uninfected subpopulations to identify the parameters that influence the stable states among the three species.

While using a stochastic individual-based model, we also wrote down the rate equations of microscopic reactions to analyse the average dynamics of our system, which resembles the population-level models like classical generalized Lotka–Volterra equations [45,46]. Although stochastic dynamics normally agrees with its corresponding deterministic system in large populations, it allows random demographic fluctuations which are important in some evolutionary scenarios, especially for extinctions of small populations [47]. Since antagonistic interactions such as those between parasites and hosts, and prey and predators, often lead to cyclic dynamics, where population sizes could drop and go through deep valleys, we are interested in how stochasticity arising from the individual level will impact the coexistence of the three species and their population composition (i.e. frequencies of infected and uninfected host individuals).

The focus of this work is not to rebuild predator–prey–parasite systems, which have been well studied in the past. Instead, we constructed a new framework to include key parameters such as reproduction costs and infection probability and analysed how these parameters can impact species coexistence. Our results provide a foundation to understand a coevolving system, which can be extended from our model by allowing those key parameters to evolve through new mutations under the interplay of ecology and evolution.

## 2. Methods

**Figure 1.**
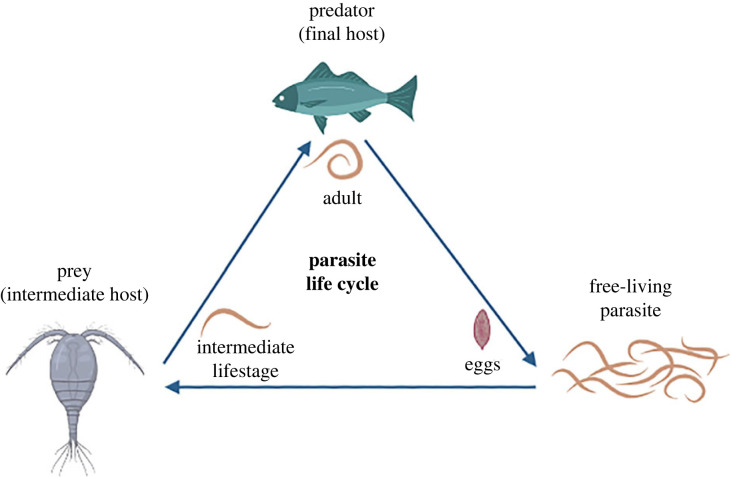
The species interactions in our model. The life cycle of a trophically transmitted parasite through two hosts under a predator–prey system.

We consider a system with three species, prey, predator and parasite (figure 1) denoted by *X*, *Y* and *Z*, respectively, in our microscopic reactions, which represent individual-level events like reproduction, death, competition, infection and predation. Since the parasite will infect an intermediate host (the prey) before being transmitted trophically to the definitive host (the predator), there are two distinct subtypes within each host species, the infected and uninfected. We denote the infected and uninfected prey populations by *X*_*I*_ and *X*_*U*_, and the infected and uninfected predator populations by *Y*_*I*_ and *Y*_*U*_. While reactions and their rates are defined at the individual level, how often, for example, reproduction or predation happens at the population level will also depend on the population abundances. For example, predation takes place in the predation rate per individual encounter multiplied by the prey and predator abundance. In the absence of the parasite, the prey and predator population interactions are described by a damped Lotka–Volterra model [48–50].

### (a) Microscopic reactions of individuals from the three species

In the absence of the other species, the prey populations can be described by the following reactions, where $g_{x}\in R^{+}$ is the reproduction rate of the prey, *r*_*x*_ ∈ [0, 1] is reproductive costs on infected prey. Here, $K\in R^{+}$ is the environmental carrying capacity of the prey population, where we assume intra-specific competition increases mortality in the population level but does not affect reproduction. While we already apply a cost of infection in reproduction, we assume that infection does not influence the competition outcome between infected and uninfected prey. However, this cost of competition can be easily implemented if necessary. We note here that infected prey offspring are uninfected [17,18].

$$\begin{array}{rl} & X_{I}\overset{g_{x}r_{x}}{\to }X_{I}+X_{U},\\ & X_{U}\overset{g_{x}}{\to }X_{U}+X_{U},\\ & X_{I}+X_{I}\overset{\frac{1}{K}}{\to }X_{I},\\ & X_{I}+X_{U}\overset{\frac{1}{K}}{\to }X_{I},\\ & X_{U}+X_{U}\overset{\frac{1}{K}}{\to }X_{U}\\ \mathrm{and}\;\; & X_{U}+X_{I}\overset{\frac{1}{K}}{\to }X_{U}. \end{array}$$

An uninfected prey encounters a free-living parasite at rate *S*, where $S\in R^{+}$. It is then infected with probability *Q*_*x*_ ∈ [0, 1]. When a parasite successfully infects the prey, the parasite is removed from the free-living parasite population. Otherwise, it remains in the free-living parasite population.

$$X_{U}+Z\overset{Q_{x}S}{\to }X_{I}$$

and

$$X_{U}+Z\overset{(1-Q_{x})S}{\to }X_{U}+Z.$$

The predation rate per individual encounter between a prey and a predator is *f*_*y*_. The reproduction efficiency of the predator by consuming is *k*_*y*_ ∈ [0, 1], which means a consumed prey gives rise to at most one predator offspring. When an uninfected prey meets an uninfected predator, we have the following reactions:

$$X_{U}+Y_{U}\overset{k_{y}f_{y}}{\to }Y_{U}+Y_{U}$$

and

$$X_{U}+Y_{U}\overset{(1-k_{y})f_{y}}{\to }Y_{U}.$$

However, when an infected prey meets an uninfected predator, the predator can be infected with probability *Q*_*y*_ ∈ [0, 1]. If the predator is successfully infected by the parasite through consumption of infected prey, the infected predator’s ability to reproduce is affected by the presence of the parasite and hence is reduced according to parameter *r*_*p*_ ∈ [0, 1]. Meanwhile, the parasite transmitted from the primary host, the prey, will reproduce within the definitive host, the predator, and have $n_{z}\in Z^{+}$ offspring per successful infection. This indicates that in our model, the free-living parasites will only be released after a parasite finishes its life cycle and reproduces in its final hosts, and thus is coupled with the success of predation.

$$X_{I}+Y_{U}\overset{r_{p}k_{y}Q_{y}f_{y}}{\to }Y_{I}+Y_{U}+n_{z}Z$$

and

$$X_{I}+Y_{U}\overset{(1-r_{p}k_{y})Q_{y}f_{y}}{\to }Y_{I}+n_{z}Z.$$

Being exposed to a parasite triggers an immune response, which is energetically costly, even in the absence of infection [12]. If the parasite is not successful in infecting the predator through trophic transmission, there is still a reproductive cost due to exposure *r*_*e*_ ∈ [0, 1]. We assume that the reproductive cost due to infection is larger than the one due to exposure. Hence, *r*_*p*_ ≤ *r*_*e*_.

$$X_{I}+Y_{U}\overset{r_{e}k_{y}(1-Q_{y})f_{y}}{\to }Y_{U}+Y_{U}$$

and

$$X_{I}+Y_{U}\overset{\left(1-r_{e}k_{y}\right)}{\to }(1-Q_{y})f_{y}Y_{U}.$$

Similarly, when an uninfected prey meets an infected predator, the infected predator can reproduce with the cost *r*_*p*_. Note that parasite reproduction already happened when this predator was infected the first time, thus is not redundantly included here.

$$X_{U}+Y_{I}\overset{r_{p}k_{y}f_{y}}{\to }Y_{I}+Y_{U}$$

and

$$X_{U}+Y_{I}\overset{(1-r_{p}k_{y})f_{y}}{\to }Y_{I}.$$

Finally, we consider the remaining possible reactions where an infected prey meets an infected predator. If the infected predator is successfully infected again by the newly encountered infected prey, its ability to reproduce is further reduced by the same parameter *r*_*p*_. The parasite transmitted by this newly encountered prey will reproduce in its final host because this is the first time this predator is infected by this individual parasite.

$$X_{I}+Y_{I}\overset{{r_{p}}^{2}k_{y}Q_{y}f_{y}}{\to }Y_{I}+Y_{U}+n_{z}Z$$

and

$$X_{I}+Y_{I}\overset{(1-{r_{p}}^{2}k_{y})Q_{y}f_{y}}{\to }Y_{I}+n_{z}Z.$$

If the parasite is unsuccessful at infecting the infected predator, we apply a reproductive cost as before by parameter *r*_*e*_. Thus, in the following reaction where the predator reproduces, *r*_*e*_ refers to the cost of exposure in this encounter and *r*_*p*_ refers to the cost of previous infection:

$$X_{I}+Y_{I}\overset{r_{p}r_{e}k_{y}(1-Q_{y})f_{y}}{\to }Y_{I}+Y_{U}$$

and

$$X_{I}+Y_{I}\overset{(1-r_{p}r_{e}k_{y})(1-Q_{y})f_{y}}{\to }Y_{I}.$$

All individuals have an intrinsic death rate, with *d*_*x*_, *d*_*y*_ and $d_{z}\in R^{+}$ denoting that for the prey, predator and parasite, respectively. While we already apply a cost of infection or exposure in reproduction, we assume the same intrinsic death for infected and uninfected individuals for the sake of simplicity. However, costs on death due to infection can be easily implemented as an extension.

$$\begin{array}{rl} & X_{I}\overset{d_{x}}{\to }0,\\ & X_{U}\overset{d_{x}}{\to }0,\\ & Y_{I}\overset{d_{y}}{\to }0,\\ & Y_{U}\overset{d_{y}}{\to }0\\ \mathrm{and}\;\; & Z\overset{d_{z}}{\to }0. \end{array}$$

We use a standard Gillespie algorithm [51,52] to perform stochastic simulations of the time evolution of the prey and predator subtypes and the parasite. See table S1 of the electronic supplementary material for the corresponding propensity equations and reactions. A summary of parameters is given in table 1.

**Table 1.** Parameter definitions.

parameter	definition
*g*_*x*_	reproduction rate of the prey
*r*_*x*_	reproductive cost on the prey due to parasite infection
*d*_*x*_	intrinsic death rate of the prey
*K*	environmental carrying capacity of the prey
*S*	scaling factor for prey–parasite interactions
*n*_*z*_	offspring per reproduction of the parasite
*d*_*z*_	intrinsic death rate of the parasite
*f*_*y*_	predation rate
*k*_*y*_	reproduction rate of the predator
*r*_*p*_	reproductive cost on the predator due to parasite infection
*r*_*e*_	reproductive cost on the predator due to parasite exposure
*d*_*y*_	intrinsic death rate of the predator
*Q*_*x*_	infection probability of parasite–prey
*Q*_*y*_	infection probability of parasite–predator

### (b) Deterministic rate equations based on microscopic reactions

We can analyse the average population dynamics by writing down the deterministic equations based on the above reactions. We introduce *x*, *y* and *z* to represent the population abundance of the prey, predator and parasite, respectively, where $x,y,z\in R^{+}$. We use subscripts to distinguish the infected and susceptible prey and predator subtypes. We let *x*_*I*_ and *x*_*U*_ denote the infected and uninfected prey subpopulations, *y*_*I*_ and *y*_*U*_ denote the infected and uninfected predator subpopulations, and *x* and *y* for the total population. Thus, *x* = *x*_*I*_ + *x*_*U*_ and *y* = *y*_*I*_ + *y*_*U*_. We derive a set of ordinary differential equations from the microscopic reactions, where $\dot{x_{I}}$, $\dot{x_{U}}$, $\dot{y_{I}}$, $\dot{y_{U}}$ and $\dot{z}$ denote the rates of change of corresponding populations with respect to time:

$$\begin{array}{rl} & \dot{x_{I}}=-\frac{(x_{I}+x_{U})x_{I}}{K}-d_{x}x_{I}+Q_{x}Sx_{U}z-f_{y}x_{I}(y_{I}+y_{U}),\\ & \dot{x_{U}}=g_{x}(r_{x}x_{I}+x_{U})-\frac{(x_{I}+x_{U})x_{U}}{K}-d_{x}x_{U}-Q_{x}Sx_{U}z-f_{y}x_{U}(y_{I}+y_{U}),\\ & \dot{y_{I}}=-d_{y}y_{I}+Q_{y}f_{y}x_{I}y_{U},\\ & \dot{y_{U}}=k_{y}f_{y}x_{U}(r_{p}y_{I}+y_{U})+(r_{e}k_{y}(1-Q_{y})-(1-r_{p}k_{y})Q_{y})f_{y}x_{I}y_{U}+(r_{p}r_{e}k_{y}(1-Q_{y})+{r_{p}}^{2}k_{y}Q_{y})f_{y}x_{I}y_{I}-d_{y}y_{U}\\ \mathrm{and}\; & \dot{z}=-Q_{x}Sx_{U}z+n_{z}Q_{y}f_{y}x_{I}(y_{I}+y_{U})-d_{z}z. \end{array}$$

## 3. Results

### (a) Equilibrium analysis for the deterministic system

We first analyse the coexistence of all three species based on our deterministic equations. When the parasite enters the predator–prey system, it must have a basic reproduction value greater than unity to become endemic. Given the parasite can only reproduce in an infected predator, we show that the parasite can always invade a predator–prey system if *S*, *Q*_*x*_, *Q*_*y*_, *z* > 0 (see §2.1 of the electronic supplementary material). We note here that a feature of the deterministic system considered is the coexistence of all species when *z* > 0.

When, *z* > 0, we have two scenarios, stable coexistence of all species or chaotic dynamics, as *t* → ∞. We can obtain the equilibria when $\dot{x_{I}}=\dot{x_{U}}=\dot{y_{I}}=\dot{y_{U}}=\dot{z}=0$ for *x*_*I*_, *x*_*U*_, *y*_*I*_, *y*_*U*_, *z* > 0. The steady state of infected prey population, ${x_{I}}^{*}$, is a solution to the following polynomial (see details in §2.2 of the electronic supplementary material):

3.1 $$\begin{array}{rl} & \Theta ((x_{I}+\Psi +Kd_{x})(K\Phi \Psi \alpha \eta x_{I}+K\Theta \Phi \Psi f_{y})-(Kg_{x}(r_{x}x_{I}+\Psi )-\Psi (x_{I}+\Psi +Kd_{x}))(K\Phi \Psi \alpha \eta -K\Theta \Phi f_{y}))=0. \end{array}$$

Here, we have functions $\Phi =\lambda x_{I}+1$, $\Psi =(d_{y}-\epsilon x_{I}-\zeta \lambda {x_{I}}^{2})/(\gamma \lambda x_{I}+\delta )$ and $\Theta =\alpha \Psi +d_{z}$, where *α* = *Q*_*x*_*S*, *β* = *Q*_*y*_*f*_*y*_, *γ* = *r*_*p*_*k*_*y*_*f*_*y*_, *δ* = *k*_*y*_*f*_*y*_, $\epsilon =(r_{e}k_{y}(1-Q_{y})-(1-r_{p}k_{y})Q_{y})f_{y}$, $\zeta =(r_{p}r_{e}k_{y}(1-Q_{y})+{r_{p}}^{2}k_{y}Q_{y})f_{y}$, *η* = *n*_*z*_*Q*_*y*_*f*_*y*_ and *λ* = *β*/*d*_*y*_. From ${x_{I}}^{*}$, we determine the remaining steady-state populations ${x_{U}}^{*}$, ${y_{I}}^{*}$, ${y_{U}}^{*}$, z* as the following:

$$\begin{array}{rl} & {x_{U}}^{*}=\frac{d_{y}-\epsilon {x_{I}}^{*}-\zeta \lambda {{x_{I}}^{*}}^{2}}{\gamma \lambda {x_{I}}^{*}+\delta },\\ & {y_{U}}^{*}=\frac{\Theta ({x_{I}}^{*}+{x_{U}}^{*}+Kd_{x})}{K\Phi \alpha \eta {x_{U}}^{*}-K\Theta \Phi f_{y}}=\frac{K\Theta g_{x}(r_{x}{x_{I}}^{*}+{x_{U}}^{*})-\Theta ({x_{I}}^{*}+{x_{U}}^{*}+Kd_{x}){x_{U}}^{*}}{K\Phi \alpha \eta {x_{I}}^{*}{x_{U}}^{*}+K\Theta \Phi f_{y}{x_{U}}^{*}},\\ & {y_{I}}^{*}=\lambda {x_{I}}^{*}{y_{U}}^{*}\\ \mathrm{and}\; & z^{*}=\frac{\eta {x_{I}}^{*}({y_{I}}^{*}+{y_{U}}^{*})}{\Theta }. \end{array}$$

**Figure 2.**
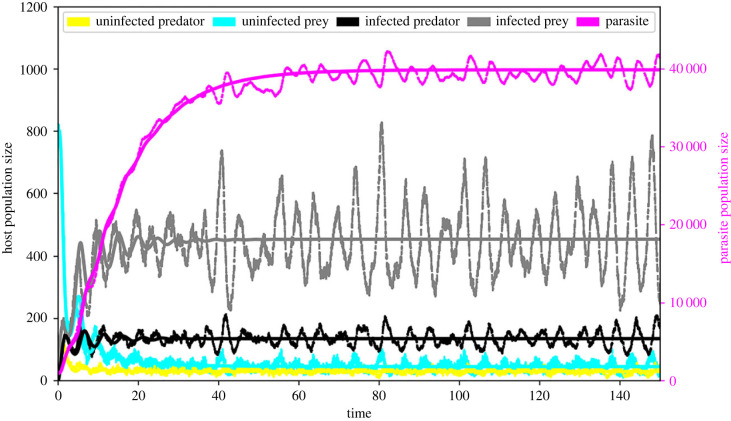
An example of population dynamics when all species coexist. The yellow colour shows the uninfected predator, the cyan colour shows the uninfected prey, the black colour shows the infected predator, the grey colour shows the infected prey and the magenta colour shows the parasites. The solid lines show the population dynamics according to the deterministic dynamics and the dashed lines according to stochastic simulations. Parameter values: *g*_*x*_ = 2, *r*_*x*_ = 1, *d*_*x*_ = 0.1, *K* = 2000, *S* = 0.0005, *n*_*z*_ = 6, *d*_*z*_ = 0.09, *f*_*y*_ = 0.01, *k*_*y*_ = 0.2, *d*_*y*_ = 1, *r*_*e*_ = 1, *r*_*p*_ = 1, *r*_*x*_ = 1, *Q*_*y*_ = 1 and *Q*_*x*_ = 1 (see electronic supplementary material, §2.3.1). Initial conditions: uninfected predator = 100, uninfected prey = 800, infected predator = 0, infected prey = 0 and parasite = 1000.

When there is no reproductive cost (*r*_*p*_ = 1, *r*_*x*_ = 1, *r*_*e*_ = 1), we observe the coexistence of all species, where our stochastic simulations fluctuate around the numerical simulations of the rate equations and converge to the analysed equilibrium when time increases (figure 2). For a complete reproductive reduction of infected predators without altering other parameters (*r*_*p*_ = 0, *r*_*x*_ = 1, *r*_*e*_ = 1), the deterministic dynamics are cyclic and the predator population can drop into deep valleys with small values but always exists. However, the small predator population can go extinct in stochastic simulations and lead to a very different outcome, i.e. the predator species vanishes first followed by the parasite and the prey species recovers to fluctuate around its own carrying capacity (electronic supplementary material, figure S9).

### (b) Patterns of parasite, predator and prey coexistence

**Figure 3.**
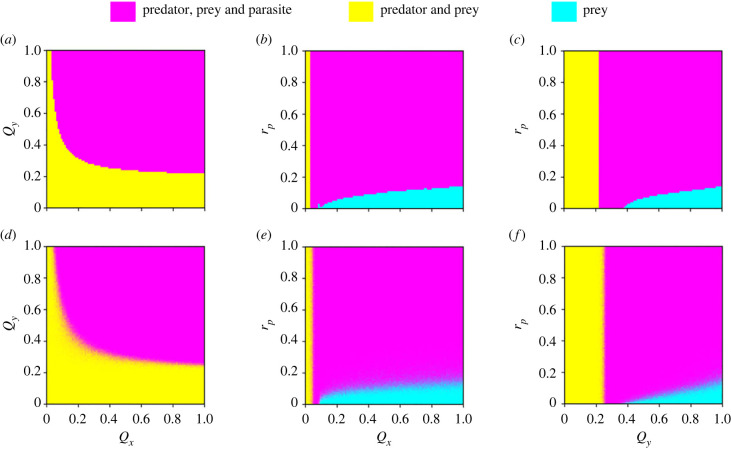
Patterns of species coexistence. (*a*–*c*) The numerical simulations of the deterministic equations and (*d*–*f*) the corresponding stochastic dynamics. In the stochastic simulations, each parameter combination displays the average outcome from 100 independent realizations, where the corresponding colour is weighted by the percentages of all possible outcomes. When the colour is closer to magenta, it means that a higher percentage of the 100 independent stochastic realizations has an outcome of three-species coexistence. Parameter values: *g*_*x*_ = 2, *d*_*x*_ = 0.1, *K* = 2000, *S* = 0.0005, *n*_*z*_ = 6, *d*_*z*_ = 0.09, *f*_*y*_ = 0.01, *k*_*y*_ = 0.2, *d*_*y*_ = 1, *r*_*e*_ = 1, *r*_*x*_ = 1 (see electronic supplementary material, §2.3.1). Panels (*a*,*d*) show *r*_*p*_ = 1; (*b*,*e*) show *Q*_*y*_ = 1; (*c*,*f*) show *Q*_*x*_ = 1. Initial conditions: uninfected predator = 100, uninfected prey = 800, infected predator = 0, infected prey = 0 and parasite = 1000. Note that *r*_*p*_ = 0 refers to high reproductive costs and *r*_*p*_ = 1 refers to the absence of reproductive costs.

We further explore all possible outcomes of species coexistence, where one (prey), two (prey and predator) or three (prey, predator and parasite) species coexist over the long term. In the absence of reproductive costs (*r*_*p*_ = 1, *r*_*x*_ = 1, *r*_*e*_ = 1), the coexistence of the three species is more likely with increasing infection probabilities of hosts (*Q*_*x*_, *Q*_*y*_) under both deterministic and stochastic dynamics (figure 3*a*,*d*, magenta). This is because parasites with complex life cycles must be efficient at infecting both trophic levels to reach maturity and reproduce. Interestingly, the three species coexist in a narrower range of infection probabilities of the predator host than of the prey host, with a threshold of *Q*_*y*_ > 0.25 and *Q*_*x*_ > 0.07, respectively. Given that the parasite needs both hosts in its life cycle, these differences could stem from the predator population being smaller than the prey population.

When considering a reproductive cost only on the infected predator (*r*_*p*_ ∈ [0, 1], *r*_*x*_ = 1), we found that the stochastic simulations still agree well with the deterministic predictions in most parameter regions (figure 3*b*,*c*,*e*,*f*). For high reproductive costs on infected predators (small *r*_*p*_), the predator population becomes very small due to increasing infections either in prey (*Q*_*x*_) or in predator (*Q*_*y*_). Consequently, we observe the extinction of predator species first followed by the parasite (figure 3, cyan). However, if the infection probability of either host is too small (small *Q*_*x*_ or small *Q*_*y*_), the parasite will not be able to be maintained in the system, which leads to a coexistence of only the prey and the predator (figure 3, yellow). The threshold to avoid parasite extinction is higher in the infection probability of the predator (*Q*_*y*_) than the infection probability of the prey (*Q*_*x*_). For a complete reduction of reproduction on the predator due to infection (*r*_*p*_ = 0), there are intermediate ranges of *Q*_*x*_ and *Q*_*y*_ values, where the three species can coexist. This range increases when the reproductive costs on infected predators decrease (increasing *r*_*p*_). We see a similar pattern if there is a reproductive cost only on the infected prey (electronic supplementary material, figure S11B,C,E,F), i.e. under a complete reduction of reproduction on the prey due to infection (*r*_*x*_ = 0), the three species coexist in an intermediate range of *Q*_*x*_ and *Q*_*y*_ values and this range increases with increasing *r*_*x*_. Finally, when there are reproductive costs both on the prey and the predator (electronic supplementary material, figure S11A,D), the coexistence of the three species only happens when the costs on both hosts are below certain thresholds.

In the border parameter regions between coexistence and extinction, while there is only one possible outcome in deterministic dynamics, both coexistence and extinction can happen in stochastic repeats under the same parameter values. This illustrates the importance of stochasticity and demographic fluctuations in species coexistence. In addition, there is a small shift between the clear border of the deterministic dynamics and the vague border of the stochastic dynamics, where there is a slightly wider region of three-species coexistence (figure 3, magenta) under the deterministic dynamics compared to the stochastic dynamics. This is likely to arise from stochastic extinction in abundance cycles under species interactions. In the deterministic dynamics, even if an abundance cycle is with large amplitudes under coexistence, i.e. small population sizes in the valleys, the species will not go extinct because the dynamics are deterministic. However, in the stochastic dynamics, such cycles with deep valleys can lead to species’ extinction at random, thus all possible outcomes can happen among independent realizations in a given parameter set. This explains why the deterministic dynamics for three-species coexistence is slightly larger than the stochastic dynamics.

### (c) Frequency of uninfected and infected host subtypes

**Figure 4.**
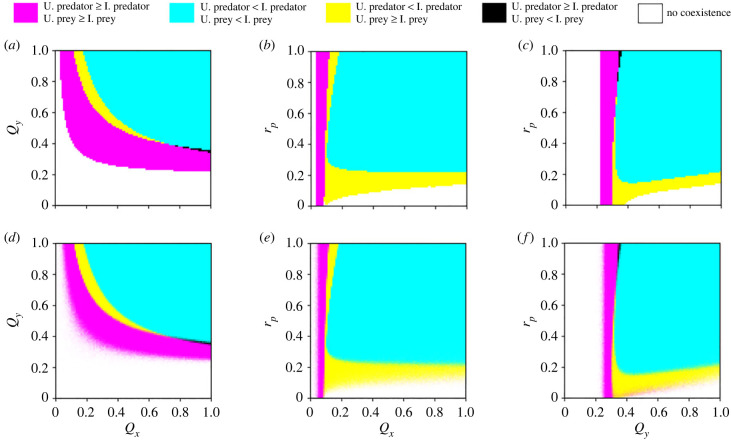
Comparing the relative frequencies of infected and uninfected prey and predators. (*a*–*c*) The numerical simulations of the deterministic equations and (*d*–*f*) the corresponding stochastic dynamics. In the stochastic simulations, each parameter combination displays the average outcome from 100 independent realizations, where the corresponding colour is weighted by the percentages of all possible outcomes. Parameter values: *g*_*x*_ = 2, *d*_*x*_ = 0.1, *K* = 2000, *S* = 0.0005, *n*_*z*_ = 6, *d*_*z*_ = 0.09, *f*_*y*_ = 0.01, *k*_*y*_ = 0.2, *d*_*y*_ = 1, *r*_*e*_ = 1, *r*_*x*_ = 1 (see electronic supplementary material, §3.2.1). Panels (*a*,*d*) show *r*_*p*_ = 1; (*b*,*e*) show *Q*_*y*_ = 1; (*c*,*f*) show *Q*_*x*_ = 1. Initial conditions: uninfected predator = 100, uninfected prey = 800, infected predator = 0, infected prey = 0 and parasite = 1000. Note that *r*_*p*_ = 0 refers to high reproductive costs and *r*_*p*_ = 1 refers to the absence of reproductive costs.

We consider the composition of infected and uninfected host subpopulations when the predator, prey and parasite coexist (figure 4). As expected, infected individuals are more frequent than their uninfected counterparts with increasing infection probabilities and a threshold of *Q*_*x*_ > 0.18 and *Q*_*y*_ > 0.36, in both the deterministic and stochastic simulations (figure 4*a*,*d*, cyan). Similarly, uninfected hosts coexist with the parasite at higher frequencies than their infected counterparts under lower infection probabilities (figure 4*a*,*d*, magenta).

Interestingly, we found that all combinations of subpopulations’ coexistence are possible, albeit at very different likelihoods. For instance, we often observe that a higher number of infected predators is combined with a higher amount of uninfected prey (figure 4, yellow). This combination happens when the parasite is more efficient at infecting the predator than the prey, with a threshold of *Q*_*x*_ = [0.12–0.69] and *Q*_*y*_ > 0.41 (figure 4*a*,*d*, yellow). If *Q*_*y*_ = 1 and *r*_*p*_ = 1, which refers to the highest infection probability of the predator without any cost, this outcome happens in a small parameter range of infection probabilities of the prey *Q*_*x*_ = [0.12–0.18] (figure 4*b*,*e*, yellow). Contrarily, with high reproductive costs on infected predators (small *r*_*p*_), this parameter space increases and we see a high frequency of uninfected prey regardless of the infection probabilities of both hosts (figure 4*b*,*c*,*e*,*f*, magenta and yellow). This suggests that the prey population can recover from infection when parasite pressure leads to few predators, which relaxes the predation pressures on the prey.

A high frequency of infected prey combined with a high frequency of uninfected predators is the least common outcome, yet an interesting one (figure 4, black). This combination of host frequencies occurs in a parameter space in which the parasite is less efficient at infecting the predator than the prey (with a threshold of *Q*_*y*_ = 0.33–0.38), combined with low reproductive costs on infected predators (*r*_*p*_ > 0.6). Due to the complex life cycle of the parasite, the predator must eat infected prey to be exposed to the parasite. When there is a high availability of infected prey, the transmission of the parasite to the predator ultimately depends on the predator’s resistance/susceptibility. This explains why, when the infection probability of the predator is high (*Q*_*y*_ = 1), we do not see the coexistence of infected prey and uninfected predators at high frequencies according to any probability of the parasite infecting the prey (figure 4; electronic supplementary material, figure S12B,E). With low reproductive costs on infected predators, the predator population recovers rapidly from infection because we assume that the parasite is not vertically transmitted to the next generation [17,18]. In addition, when the predator is less efficient at consuming the prey, the predator population is unable to recover from infection, regardless of the type of prey that is available.

**Figure 5.**
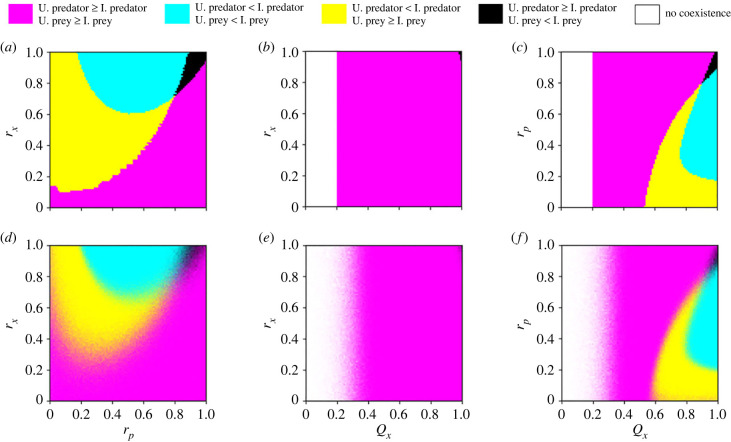
The relative frequencies of infected and uninfected prey and predators under *Q*_*y*_ = 0.35. (*a*–*c*) The numerical simulations of the deterministic equations and (*d*–*f*) the corresponding stochastic dynamics. In the stochastic simulations, each parameter combination displays the average outcome from 100 independent realizations, where the corresponding colour is weighted by the percentages of all possible outcomes. Parameter values: *g*_*x*_ = 2, *d*_*x*_ = 0.1, *K* = 2000, *S* = 0.0005, *n*_*z*_ = 6, *d*_*z*_ = 0.09, *f*_*y*_ = 0.01, *k*_*y*_ = 0.2, *d*_*y*_ = 1, *r*_*e*_ = 1 (see electronic supplementary material, §3.2.1). Panels (*a*,*d*) show *Q*_*x*_ = 1; (*b*,*e*) show *r*_*p*_ = 1; (*c*,*f*) show *r*_*x*_ = 1. Initial conditions: uninfected predator = 100, uninfected prey = 800, infected predator = 0, infected prey = 0 and parasite = 1000. Note that *r*_*p*_ = 0 and *r*_*x*_ = 0 refer to high reproductive costs and *r*_*p*_ = 1 and *r*_*x*_ = 1 refer to the absence of reproductive costs.

To further investigate the conditions leading to a high frequency of infected prey and uninfected predators, we tested the effects of the parameters when *Q*_*y*_ = 0.35, which is in the critical parameter range resulting in these frequencies of host subpopulations. We found that the variation in the probability of prey infection (*Q*_*x*_) and reproductive costs on infected prey (*r*_*x*_) become practically irrelevant (figure 5*b*,*e*). Instead, this outcome is mainly driven by low reproductive costs on infected predators (large *r*_*p*_). This suggests that the probability of the parasite infecting the prey is ultimately mediated by the predation pressures which are, in turn, mediated by the parasite-mediated selection, hence revealing an indirect parasite effect.

Again, we observe a difference between the deterministic and stochastic results especially at the border regions between the yellow and magenta areas in figure 5*a*,*d*. The definitions of yellow and magenta areas in figure 5 differ only in the order of the subpopulation sizes of uninfected and infected predators. At low *r*_*x*_ and *r*_*p*_ values, the subpopulation sizes of uninfected and infected predator populations are relatively close in deterministic dynamics (see electronic supplementary material, figure S10). Thus, given the nature of the stochastic dynamics, we expect some variation in the classification between yellow and magenta in this parameter space.

## 4. Discussion

Growing evidence suggests that parasites mediate trophic interactions [41,42] and that predators regulate host–parasite interactions [53,54]. Yet, the interplay of those dynamics remains understudied, particularly when parasites have complex life cycles [16]. To address this knowledge gap, we implemented an individual-based model of a complex predator–prey–parasite system, where the parasite is transmitted trophically from infected prey to predators. We showed that the infection probabilities of both host species and parasite virulence (reproductive costs on infected hosts) determine the coexistence of the three species (figure 3; electronic supplementary material, figure S11). We compared our deterministic simulation results with stochastic dynamics. In almost all parameter regimes, the stochastic simulations agree well with deterministic predictions. In the border regions (e.g. between coexistence and extinction in the parameter space), while only one outcome is possible under deterministic dynamics for each parameter set, both coexistence and extinction can happen in different stochastic realizations. This demonstrates the relevance of demographic fluctuations in species coexistence. When decomposing the host populations into uninfected and infected subpopulations, we found multiple stable states of the three species which are determined by the infection probabilities and parasite virulence across the trophic levels (figure 4).

Generally, we show that the three species are more likely to coexist with increasing infection probabilities of both hosts because parasites with complex life cycles need to infect both trophic levels to develop and reproduce [55,56]. Interestingly, our results further show that, for the persistence of the parasite, the probability of infecting the predator is more critical than the probability of infecting the prey (figure 3*a*,*d*). Given that the parasite needs both hosts in its life cycle, these differences could stem from the different population sizes of the predator and prey. We suggest that the parasite needs to be more efficient at infecting the predator because the encounter probabilities between uninfected predators and infected prey are lower than those between free-living parasites and uninfected prey. A non-excluding alternative explanation is that the availability of infected or uninfected prey becomes less relevant when the parasite is inefficient at infecting the predator, i.e. with a low probability of infecting the predator. Our finding that infecting the predator is more critical for the persistence of the parasite could possibly explain why selection leads to the manipulation of intermediate hosts by trophically transmitted parasites [19,57]. When the likelihood of finding a predator is low, the manipulation of the intermediate host (the prey) enhances parasite fitness by increasing the probability of infected prey being more eaten than non-infected prey. Recent theoretical work demonstrates that strong host manipulation can stabilize community dynamics and allow a trophically transmitted parasite to coexist with its intermediate and final hosts [58].

Previous deterministic models of parasite dynamics within simple communities predict that parasites should generally go extinct before their hosts [59]. Here, using complex communities, we show similar patterns whereby, in most cases, the parasite goes extinct before the predator, but the prey always survives. Our simulations, however, reveal that both the predator and the parasite can go extinct with high reproductive costs on either host combined with medium to high probabilities of the parasite infecting either host (figure 3; electronic supplementary material, figure S11, cyan). This result is consistent with theoretical work on a predator–prey–parasite system where the parasite infects only the prey, showing that when the prey population is infected at a low rate, and the predator has low reproductive potential, the extinction of both the predator and the parasite can happen [60]. We suggest, however, that the underlying mechanisms are different when considering a parasite that must infect both trophic levels. In our simulations, indeed we show that the extinction of both predator and parasite happens with low reproductive potential of the predator, however, in combination with medium to high probabilities of the parasite infecting both hosts (figure 3*b*,*c*,*e*,*f*, cyan). This pattern emerges because, in that parameter space, the predator population size is small. Furthermore, our results indicate that, due to the parasite’s complex life cycle, the fitness of one host (estimated in the form of reproductive success) can change with the probability of the other host getting infected. These complex interactions are reflected in our simulations, where the likelihood of predator–parasite co-extinction increases with high infection probabilities of the predator combined with high reproductive costs on infected prey (electronic supplementary material, figure S11C,F, cyan), as well as with high infection probabilities of the prey combined with high reproductive costs on infected predators (figure 3*b*,*e*, cyan). These results illustrate an indirect parasite effect on predator–prey dynamics and highlight the ecological relevance of complex species interactions.

To better understand the dynamics of the parasite’s demography, we decoupled the predator and prey populations into uninfected and infected subpopulations when the parasite coexists with the predator and the prey at a stable equilibrium. The most common outcome of our simulations reveals a high frequency of infected predators coexisting with a high frequency of infected prey (figure 4, cyan). As expected, the frequencies of infected hosts are higher with increasing infection probabilities and are lower with decreasing infection probabilities. Intuitively, due to the parasite’s complex life cycle, we expected the frequencies of infected and uninfected predator subpopulations to match with those of the prey. Yet, although less common, in some parameter spaces, this is not the case. Particularly, with high reproductive costs on infected predators there is a high frequency of uninfected prey, regardless of the infection probabilities of both hosts (figure 4*b*,*c*,*e*,*f*, magenta and yellow). Overall, we see that the prevalence of infection in the prey population is mostly driven by the reproductive costs on infected predators (figure 4*b*,*c*,*e*,*f*) rather than the reproductive costs on infected prey (electronic supplementary material, figure S12A–F). As the reproductive costs on infected predators increase, the predator population size drops, hence relaxing the predation pressures on the prey. Consequently, the prey population size recovers mostly with uninfected prey, illustrating an indirect parasite effect on the prey through the predator.

We found that the least likely combination of host subpopulations is a high frequency of infected prey coexisting with a high frequency of uninfected predators (figure 4, black). While this outcome is interesting, it is rare and happens in a narrow parameter space of infection probability of the predator *Q*_*y*_ = [0.33–0.38], combined with low reproductive costs on infected predators *r*_*p*_ > 0.6. Given that our model does not assume vertical transmission of the parasite to the prey or predator offspring [17,18], we expected the prey population to recover from infection in the absence of reproductive costs on infected prey. Surprisingly, the likelihood of observing a higher frequency of infected prey coexisting with a higher frequency of uninfected predators increases with lowering reproductive costs on infected prey (figure 5, black). We suggest that the prey recovers slower from infection due to strong predation pressures in this parameter space, particularly as the infection probability is higher on the prey than on the predator. This result is coherent with the healthy herd hypothesis, which proposes that the dynamics of prey–parasite interactions are determined by the predator population size and parasite virulence [53]. Also, experimental evidence shows that the parasite’s impact on the population size of an intermediate consumer is obscured by that of the predator when predators are abundant [29], which is a characteristic we found often in our system.

In natural systems, parasites can be virulent, and yet sometimes do not cause significant changes in host density [61]. For instance, Duffy & Hall theoretically tested the effects of two parasites, the bacterium *Spirobacillus cienkowskii* and the yeast *Metschnikowia bicuspidata*, on the common freshwater invertebrate *Daphnia dentifera* [61]. While both parasites are known to be virulent, only bacterial epidemics led to significant changes in the host density. Interestingly, they found that the rapid evolution of host resistance to the yeast parasite combined with predation mostly on infected hosts decreased the prevalence of infection and minimized host density decline [61]. Further theoretical work on eco-epidemiological models, where the parasite infects the prey population, showed that the predators’ preference for uninfected and infected prey individuals plays a significant role in the system dynamics and stability [32,60]. We suggest that these dynamics may be different in systems where the parasite is transmitted trophically because the infection probabilities and parasite virulence can vary across trophic levels [62].

Based on our results, we propose that the combination of host subpopulations in different trophic levels may have important ecological consequences. For instance, if a predator evolves the capacity to eat preferentially uninfected prey, high predation pressures will result in a prey population of highly abundant infected individuals [53]. The resulting high incidence of infected prey may lead to a higher likelihood of transmission of the parasite to other predators that lack a discriminative feeding behaviour. We suggest that the transmission of these parasites to other predator species will ultimately depend on multiple factors such as parasite virulence and the immunity of those hosts. Similarly, a high incidence of infected predators or a high incidence of infected prey may lead to trophic cascades [40] with potentially important consequences at the community level [41,42].

Overall, we demonstrate that the interplay of direct and indirect parasite effects along a food web is a common driver of the prevalence of infections. The parasite can impact the host population directly through the reproductive costs resulting from infection, or indirectly via changing the demography of the interacting species, i.e. the predator or the prey of the host. The combination of infection probabilities and reproductive costs on infected hosts of different trophic levels determines the internal stability of the system because parasites change the demography of the host populations (a direct parasite effect) and, consequently, predator–prey dynamics (an indirect parasite effect). Moreover, by analysing the dynamics of different host subpopulations, we provide quantitative data on the prominent features of a multi-species system driving the dynamics of a trophically transmitted parasite. In some parameter spaces, we show that both extinction and coexistence can happen in different stochastic realizations. Although we focus on a static predator–prey–parasite system, the stochastic uncertainties might lead to completely different evolutionary outcomes when mutations and coevolution happen. We propose that integrating the evolution of host resistance and parasite infectivity in complex models may reveal important aspects of these complex interactions because the rates of infection vary according to host–parasite coevolution. Particularly, hosts from different trophic levels may coevolve with the parasite differently, according to their population sizes and parasite virulence. Yet, our study provides new insights into the mechanisms driving the dynamics of trophically transmitted parasites, with important implications for forecasting and managing the spread of diseases in natural ecosystems.

## Data Availability

All code associated with this paper are available online at https://github.com/WeiniHuangBW/Predator-Prey-Parasite. The data are provided in electronic supplementary material [63].
